# Atypical congenital toxoplasmosis presenting with neonatal jaundice and central nervous system involvement: a case report and therapeutic challenges to limited access to first-line anti-toxoplasma medications

**DOI:** 10.3389/fped.2026.1874973

**Published:** 2026-06-25

**Authors:** Li Wang, Kaiwei Ding, Shuai Yu, Zheng Guo, Yongjiao Wang, Lingkong Zeng, Wenhao Yuan

**Affiliations:** 1 Department of Neonatology, Wuhan Children’s Hospital (Wuhan Maternal and Child Healthcare Hospital), Tongji Medical College, Huazhong University of Science and Technology, Wuhan, China; 2 Department of Cardiothoracic Surgery, Wuhan Children’s Hospital (Wuhan Maternal and Child Healthcare Hospital), Tongji Medical College, Huazhong University of Science and Technology, Wuhan, China; 3 Department of Ophthalmology, Wuhan Children’s Hospital (Wuhan Maternal and Child Healthcare Hospital), Tongji Medical College, Huazhong University of Science and Technology, Wuhan, China; 4 Department of Radiology, Wuhan Children’s Hospital (Wuhan Maternal and Child Healthcare Hospital), Tongji Medical College, Huazhong University of Science and Technology, Wuhan, China

**Keywords:** case report, chorioretinitis, congenital toxoplasmosis, hearing loss, metagenomic next-generation sequencing, neonatal jaundice, toxoplasma gondii

## Abstract

**Background:**

Congenital toxoplasmosis (CT) is a vertically transmitted infection with a variable clinical spectrum, ranging from asymptomatic infection at birth to severe neurological and ocular sequelae. While the classic triad of hydrocephalus, intracranial calcifications, and chorioretinitis is well characterized, isolated neonatal hyperbilirubinemia as the initial presenting feature is uncommon and may delay diagnosis. We report a case of CT in a Chinese neonate who presented with jaundice and was subsequently found to have subclinical active chorioretinitis, cerebral edema, and bilateral central auditory pathway dysfunction. The case also illustrates therapeutic challenges related to the availability of first-line anti-parasitic agents.

**Case presentation:**

A 9-day-old term male infant was admitted for persistent jaundice. He was born at 39 ^+^ ^4^ weeks' gestation, with a prenatal history notable only for maternal cat exposure and treated hypothyroidism. Initial serological testing at the referring hospital revealed positive Toxoplasma gondii IgM and IgG. After transfer, two consecutive blood metagenomic next-generation sequencing (mNGS) tests detected T. gondii DNA (reads: 6 and 7). The combination of negative first-trimester maternal serology, postpartum maternal IgM/IgG positivity, neonatal IgM positivity, and repeated detection of T. gondii DNA in neonatal blood strongly supported congenital toxoplasmosis. Cerebrospinal fluid (CSF) analysis showed pleocytosis and elevated protein, while CSF mNGS was negative, possibly reflecting low pathogen burden or compartmentalized infection. Further evaluation demonstrated bilateral active chorioretinitis on fundoscopic examination, abnormal brainstem auditory evoked potentials consistent with bilateral central auditory pathway dysfunction, and brain MRI showing cerebral edema with punctate hemorrhages. Due to initial unavailability of pyrimethamine, azithromycin followed by trimethoprim-sulfamethoxazole was administered; however, no clear improvement in CSF inflammatory indices was observed during this period. After initiation of standard therapy with pyrimethamine, sulfadiazine, and folinic acid, the patient demonstrated rapid clinical improvement and radiological resolution of brain lesions on follow-up MRI, with marked improvement of chorioretinal scars.

**Conclusions:**

Clinicians should consider congenital toxoplasmosis in neonates with unexplained jaundice, even in the absence of classic clinical manifestations. Comprehensive multi-organ evaluation, including neuroimaging, ophthalmologic examination, and auditory testing, is essential for early disease characterization. Standard pyrimethamine–sulfadiazine–folinic acid therapy may be associated with better clinical and radiological outcomes and should be used when available. Long-term multidisciplinary follow-up is necessary to monitor potential sequelae.

## 1 Background

Congenital toxoplasmosis (CT) is a globally distributed parasitic infection caused by the obligate intracellular protozoan *Toxoplasma gondii* (1). Humans acquire infection primarily through ingestion of undercooked meat containing tissue cysts, consumption of water or food contaminated with oocysts shed by felids, or transplacental transmission from mother to fetus (2). The global incidence of CT is estimated at approximately 1.5 cases per 1,000 live births, with considerable geographic variation reflecting differences in maternal seroprevalence, dietary habits, and screening policies (3, 4). While nationwide epidemiological data for China remain limited, a recent national cross-sectional study of hospitalized children with congenital TORCH infections reported that CT accounted for only 2.89% of cases, yet was associated with the longest length of hospital stay among TORCH infections (5).

The clinical manifestations of CT are determined by the timing of maternal infection, parasite virulence, and host immune response (6). Approximately 60%–80% of infected neonates are asymptomatic at birth but remain at substantial risk for late-onset ocular and neurological sequelae, particularly chorioretinitis and developmental delay (7, 8). Symptomatic newborns may present with the classic triad of chorioretinitis (9%–31%), intracranial calcifications, and hydrocephalus, as well as other findings such as jaundice, hepatosplenomegaly, rash, anemia, and thrombocytopenia (9). Isolated neonatal jaundice as the primary presenting sign, without other stigmata of congenital infection, is relatively uncommon and may be mistaken for more prevalent causes of hyperbilirubinemia, such as hemolytic disease or breast milk jaundice (10).

Herein, we describe a detailed case of CT in a term Chinese neonate whose initial presentation was limited to jaundice. Subsequent systematic evaluation revealed subclinical but significant retinal, cerebral, and auditory involvement. The case also highlights the practical challenges of managing CT in regions where first-line anti-parasitic agents are not immediately available, and it illustrates the clinical improvement observed after transitioning to the standard pyrimethamine-sulfadiazine-folinic acid regimen. We discuss this case in the context of recent advances in the diagnosis, treatment, and long-term follow-up of CT.

## 2 Case presentation

### 2.1 Perinatal history and initial clinical course

A 9-day-old male infant was transferred to the Neonatal Intensive Care Unit (NICU) at Wuhan Children's Hospital for further evaluation and management of persistent neonatal jaundice. The infant was born at 39^+4^ weeks' gestation via cesarean section for maternal request at a local county hospital. The mother was a 19-year-old primigravida with a history of hypothyroidism diagnosed prior to pregnancy and well controlled on levothyroxine. She reported no febrile illness, rash, or lymphadenopathy during gestation but disclosed contact with a domestic cat throughout the pregnancy. Prenatal care included routine obstetric ultrasounds, which were reported as normal with no evidence of fetal anomalies or intracranial calcifications. First-trimester maternal TORCH serology, performed at approximately 12 weeks' gestation, was negative for *T. gondii* IgM and IgG antibodies. No repeat TORCH testing was performed later in pregnancy.

The infant was delivered in good condition with a birth weight of 3.0 kg (approximately 25th percentile), length of 50 cm (25th percentile), and head circumference of 34 cm (50th percentile). Apgar scores were 9 and 10 at 1 and 5 min, respectively. The neonatal course was initially unremarkable except for the onset of jaundice within the first 24 h of life. At the local hospital, total serum bilirubin (TSB) peaked at 405.8 μmol/L (23.7 mg/dL), prompting initiation of intensive phototherapy. As part of the evaluation for early-onset jaundice, a TORCH panel was performed and unexpectedly returned positive for *T. gondii* IgM (14.0 AU/mL) and IgG (>400 IU/mL). Given the suspicion of congenital toxoplasmosis, the infant was referred to our tertiary center on day 9 of life (DOL 9).

Upon admission to our NICU, the infant was clinically stable with only mild residual jaundice (transcutaneous bilirubin 6.8 mg/dL). He was active, fed well with formula, and exhibited normal vital signs. Physical examination was notable only for subtle icterus; there was no hepatosplenomegaly, skin rash, petechiae, microcephaly, or neurological abnormalities. Neuromuscular assessment revealed normal tone, symmetric primitive reflexes (Moro, suck, grasp), and no focal deficits. Ballard score corresponded to a gestational age of 39 weeks. Laboratory evaluation for pathologic jaundice revealed: mother and infant both blood type O, Rh-positive, with a negative direct Coombs test; normal G6PD screening; hemoglobin 145 g/L with an unremarkable peripheral blood smear; normal ALT, AST, and direct bilirubin, with only a mildly elevated total bile acid (12.3 µmol/L, reference 0–10 µmol/L) without cholestasis; normal C-reactive protein and procalcitonin; normal urinalysis and urine culture; normal blood amino acid, acylcarnitine, and urine organic acid screening; and normal thyroid function (TSH, T3, T4). The infant responded appropriately to intensive phototherapy. These findings excluded common pathologic causes, including isoimmune hemolysis, G6PD deficiency, red cell membrane disorders, hepatic dysfunction, systemic infection, urinary tract infection, inborn errors of metabolism, breast milk jaundice, and congenital hypothyroidism. The infant was placed in an incubator for thermoregulation and monitored closely.

### 2.2 Laboratory confirmation of toxoplasma gondii infection

To confirm the diagnosis, repeat serological testing for *T. gondii* was performed at our hospital laboratory using a chemiluminescence immunoassay. The results showed markedly elevated *T. gondii* IgM (399.58 IU/mL; reference <8) and IgG (241.24 IU/mL; reference <10). Concurrent maternal serum obtained postpartum was also positive for both *T. gondii* IgM and IgG. Combined with the negative first-trimester maternal serology, these findings were consistent with maternal seroconversion after the first trimester. The negative first-trimester screen indicated that maternal seroconversion likely occurred in the late second or third trimester.

Given the potential for false-positive IgM results in neonates, we employed blood metagenomic next-generation sequencing (mNGS) to directly detect *T. gondii* nucleic acid. The initial blood mNGS detected *T. gondii* with 6 unique sequence reads and a relative abundance of 100%. A repeat blood mNGS one week later again identified T. gondii with 7 reads, confirming persistent parasitemia. These molecular findings, in conjunction with the serological data, established a definitive diagnosis of congenital toxoplasmosis. At the time of diagnosis, targeted Toxoplasma PCR was not routinely available in our center; therefore, blood mNGS was used as an alternative molecular diagnostic approach.

### 2.3 Assessment of central nervous system involvement

To evaluate possible central nervous system (CNS) involvement, a lumbar puncture was performed early in the hospital course under aseptic conditions. The opening pressure was not measured due to technical limitations. Cerebrospinal fluid (CSF) analysis revealed a nucleated cell count of 31 × 10^6^/L with a lymphocytic predominance (71% lymphocytes, 29% monocytes), a positive Pandy's test for globulin, and an elevated protein concentration of 0.86 g/L (reference range 0.15–0.45 g/L). CSF glucose was normal. Notably, CSF mNGS was negative for *T. gondii* and other pathogens. Gram stain and bacterial culture were also negative.

Alternative causes of neonatal CNS inflammation, including bacterial meningitis, systemic bacterial infection, congenital CMV infection, HSV infection, syphilis, and metabolic disorders, were clinically evaluated and not supported by laboratory or imaging findings. Given the CSF pleocytosis and elevated protein, CNS involvement secondary to congenital toxoplasmosis was strongly suspected, despite the negative CSF mNGS. The discrepancy between blood and CSF molecular findings may reflect a low parasite burden within the CSF compartment or the sequestered nature of the infection within brain parenchyma. Repeat lumbar punctures performed later in the hospitalization showed persistent pleocytosis (34 and 36 × 10^6^/L, respectively) and elevated protein (0.79 and 0.80 g/L), indicating ongoing CNS inflammation.

### 2.4 Systematic screening for multisystem sequelae

Ophthalmologic Examination: Wide-field digital retinal imaging (RetCam) was performed after pupillary dilation. The examination revealed no vascular tortuosity or dilation in the posterior pole of either eye. However, a gray-white chorioretinal lesion was identified in the nasal superior quadrant of the right eye, and a similar lesion was noted in the temporal retina of the left eye. There was no associated vitritis or hemorrhage. These findings were consistent with bilateral active toxoplasmic chorioretinitis (Figure 1).

**Figure 1:**
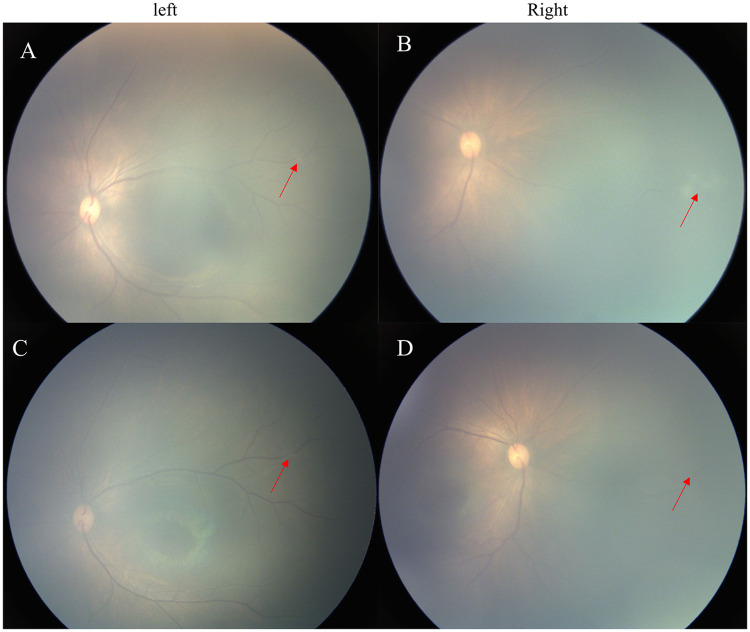
Fundus photographs demonstrating bilateral chorioretinitis before and after treatment. **(A)** Left eye at diagnosis: a well-demarcated gray-white necrotizing chorioretinal lesion was observed at the temporal vascular arcade (red arrow). **(B)** Right eye at diagnosis: a similar active gray-white chorioretinal lesion was noted in the inferonasal retina (red arrow). **(C)** Left eye after treatment: marked regression of the lesion with residual scarring (red arrow). **(D)** Right eye after treatment: significant resolution of the lesion with residual hyperpigmentation and chorioretinal scarring (red arrow).

Audiologic Evaluation: Brainstem auditory evoked potentials (BAEP) were obtained shortly after admission. Monaural stimulation with 105 dB nHL click stimuli was delivered to each ear separately. Waves I, III, and V were identifiable bilaterally, and the absolute latencies of waves I, III, and V were within normal limits in both ears. The interpeak latency between waves I and III was also normal bilaterally. However, the I-V and III-V interpeak latencies were prolonged on both sides, indicating bilateral central auditory pathway dysfunction, possibly involving the upper pons to midbrain, which may be associated with congenital toxoplasmosis (Figure 2).

**Figure 2:**
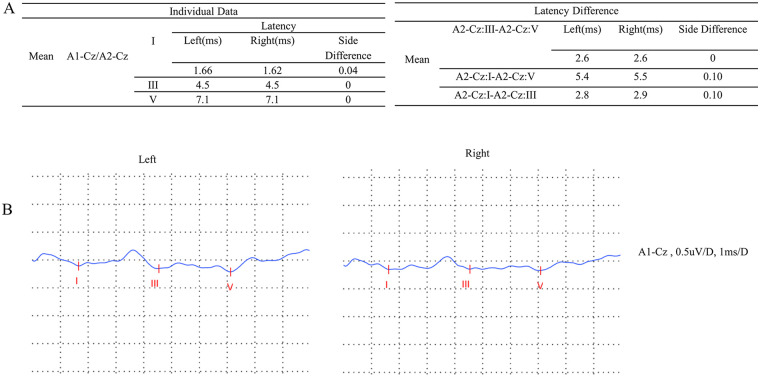
Brainstem auditory evoked potentials (BAEP) findings. **(A)** BAEP at diagnosis: prolonged I–V interpeak latency is observed bilaterally (right: 5.5 ms; left: 5.4 ms; normal [5.05 ± 0.09] ms), indicating central auditory pathway impairment. **(B)** Representative waveform illustrating delayed neural conduction along the central auditory pathway, while peripheral auditory function appears preserved.

Neuroimaging: Cranial computed tomography (CT) without contrast, performed on admission, showed no evidence of intracranial calcifications, hydrocephalus, or mass effect. However, given the CSF abnormalities, brain magnetic resonance imaging (MRI) with diffusion-weighted imaging (DWI) was obtained subsequently. The MRI demonstrated patchy, ill-defined areas of abnormal signal in the subcortical white matter of the bilateral frontal and parietal lobes. These lesions appeared hypointense on T1-weighted and T2-FLAIR images with multiple punctate foci of hyperintensity on T1-weighted images, suggestive of petechial hemorrhages. There was no ventriculomegaly, mass effect, or restricted diffusion. The findings were interpreted as cerebral edema with microhemorrhages, suggestive of inflammatory CNS involvement associated with congenital toxoplasmosis (Figure 3). A comprehensive timeline summarizing these diagnostic events is shown in Figure 4.

**Figure 3:**
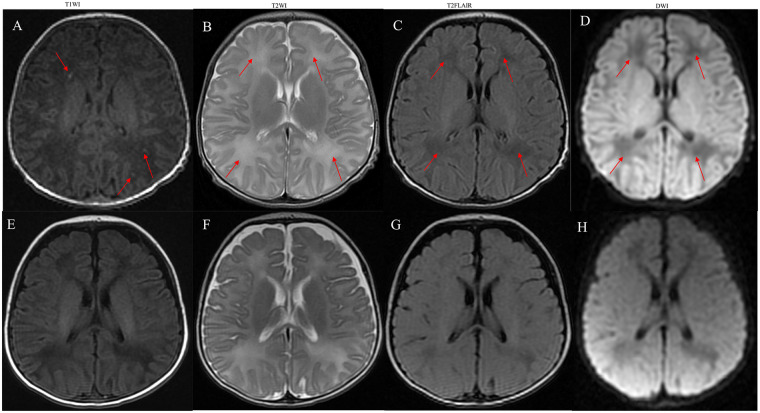
Brain MRI findings at diagnosis and follow-up. **(A–D)** MRI at diagnosis. **(A)** Axial T1-weighted image (T1WI) showing patchy hypointense signals in the subcortical white matter of the bilateral frontal and parietal lobes (arrows). **(B)** Axial T2-weighted image (T2WI) demonstrating corresponding hyperintense lesions with multiple punctate hyperintense foci within the affected regions (arrows). **(C)** Axial fluid-attenuated inversion recovery (FLAIR) image showing hypointense signals in the same regions (arrows). **(D)** Diffusion-weighted imaging (DWI) revealing hypointense signals without evidence of restricted diffusion (arrows). **(E–H)** Follow-up MRI after treatment: previously observed abnormal signals in the bilateral frontal and parietal subcortical white matter have completely resolved, with no residual lesions detected. These findings are suggestive of reversible inflammatory changes rather than permanent structural injury.

**Figure 4:**
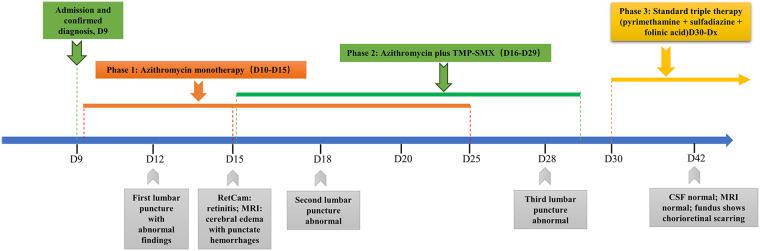
Treatment timeline and corresponding clinical response. Graphical timeline illustrating sequential therapeutic interventions and associated clinical outcomes. Phase 1: azithromycin monotherapy, with no significant improvement in clinical or laboratory parameters. Phase 2: combination therapy with azithromycin and trimethoprim-sulfamethoxazole (TMP–SMX), with minimal response. Phase 3: standard therapy with pyrimethamine, sulfadiazine, and folinic acid, resulting in marked clinical improvement. Clinical outcomes included normalization of cerebrospinal fluid parameters, complete resolution of neuroimaging abnormalities, and regression of chorioretinal lesions. The graphical timeline was included to facilitate visualization of sequential therapeutic adjustments and corresponding clinical responses.

### 2.5 Therapeutic management and challenges

The diagnosis of CT with CNS, ocular, and auditory involvement was established, and anti-parasitic therapy was urgently indicated. According to current CDC and AAP recommendations, the recommended first-line regimen for infants with symptomatic congenital toxoplasmosis consists of pyrimethamine (loading dose 2 mg/kg/day divided twice daily for 2 days, followed by 1 mg/kg/day once daily, with dosing frequency reduced later in the course), sulfadiazine (100 mg/kg/day divided twice daily), and folinic acid (10 mg three times weekly) for a total of 12 months (8, 11, 12).

However, at the time of diagnosis, pyrimethamine was not readily available in our hospital pharmacy or through local suppliers in Wuhan. After consultation with the clinical pharmacy and pediatric infectious disease services, alternative treatment strategies were considered based on published literature and expert opinion.

Initial Therapy with Azithromycin: Azithromycin has demonstrated *in vitro* and *in vivo* activity against *T. gondii* tachyzoites and tissue cysts, and several case reports and small series have suggested its potential utility in CT (13, 14). Oral azithromycin was initiated at a dose of 10 mg/kg once daily. The patient tolerated the medication well without adverse effects. However, after 6 days of azithromycin monotherapy, a repeat lumbar puncture revealed a slight increase in CSF nucleated cells (from 31 to 34 × 10^6^/L) and persistently elevated protein, suggesting limited early improvement during the observed treatment period. Azithromycin was therefore discontinued.

Second-Line Therapy with Trimethoprim-Sulfamethoxazole (TMP-SMX): A network meta-analysis of treatments for ocular toxoplasmosis suggested that TMP-SMX may be an effective alternative to pyrimethamine-sulfadiazine (15). After obtaining written informed consent from the parents for off-label use in a neonate under 2 months of age, oral TMP-SMX (dosed at approximately 10–12 mg/kg/day of the trimethoprim component, divided twice daily) was initiated, in combination with a second course of azithromycin (10 mg/kg/day). The azithromycin was discontinued after 10 days, while TMP-SMX was continued. Despite 13 days of TMP-SMX therapy, a third CSF analysis showed persistent pleocytosis (36 × 10^6^/L) and elevated protein (0.80 g/L). The persistent CSF abnormalities suggested that the alternative regimen might have provided limited control of CNS inflammatory activity in this patient.

Transition to Standard Therapy: After extensive efforts, the family was able to obtain pyrimethamine from an external source. Following discharge from the initial hospitalization with close outpatient follow-up, the patient was transitioned to the standard triple regimen: pyrimethamine (0.5 mg/kg/dose twice daily for the first 4 days, followed by 0.5 mg/kg once daily), sulfadiazine (50 mg/kg/dose twice daily), and folinic acid (10 mg three times weekly). The infant tolerated this regimen without significant adverse effects, although mild transient neutropenia was noted and managed with folinic acid dose adjustment.

### 2.6 Follow-up and outcome

The patient was re-admitted electively at 6 weeks of age (after approximately 2 weeks of definitive therapy) for a comprehensive reassessment. Clinically, he was thriving with a weight of 5.09 kg (50th percentile) and normal vital signs. The jaundice had completely resolved, and there were no neurological deficits on examination.

Repeat investigations demonstrated clear evidence of treatment response:

- CSF analysis: The nucleated cell count had normalized to 7 × 10^6^/L (from a peak of 36), and the protein concentration had decreased to 0.69 g/L, approaching the upper limit of normal.
- Brain MRI: Follow-up MRI showed complete resolution of the previously noted white matter signal abnormalities and petechial hemorrhages (Figure 3). No new lesions or calcifications were observed.
- Ophthalmologic examination: Serial fundus examinations during this period demonstrated progressive regression of the retinal lesions, with decreased size and activity of the chorioretinal scars (Figure 1). Topical tobramycin was discontinued.
- Audiology: A follow-up BAEP was scheduled for 3 months of age as per protocol.

The patient was discharged on the standard triple regimen with plans for a total treatment duration of 12 months. A multidisciplinary follow-up program was established, including periodic assessments by neonatology, pediatric infectious disease, ophthalmology, audiology, and developmental pediatrics. Table 1 summarizes the key laboratory and imaging findings before and after definitive therapy.

**Table 1:** Summary of Key laboratory and imaging findings before and after definitive therapy.

Parameter	Initial Findings (Day 12–20 of Life)	Follow-up Findings (6 Weeks of Life)
Blood *T. gondii* mNGS	Positive (Reads: 6 and 7)	Not repeated
CSF Nucleated Cells (×10^6^/L)	31 → 34 → 36	**7**
CSF Protein (g/L)	0.86 → 0.79 → 0.80	**0.69**
CSF mNGS for *T. gondii*	Negative ×2	Not repeated
Brain MRI	Cerebral edema with punctate hemorrhages in subcortical white matter	**Complete resolution** of abnormalities
Fundus Examination	Bilateral active gray-white retinal lesions	**Significant regression** of lesions, transition to scars
BAEP	Prolonged I-V and III-V interpeak latencies	Pending follow-up

mNGS, metagenomic next-generation sequencing; CSF, cerebrospinal fluid; MRI, magnetic resonance imaging; BAEP, brainstem auditory evoked potential.

Bold values indicate findings after definitive therapy: ‘Complete resolution’ denotes complete resolution of previously observed abnormal signals; ‘Significant regression’ denotes transition from active acute retinal lesions to scar tissue.

## 3 Discussion and literature review

We present a detailed case of congenital toxoplasmosis in a term neonate whose initial clinical manifestation was isolated hyperbilirubinemia, a presentation that is relatively uncommon and may lead to diagnostic delays if not recognized. The subsequent identification of subclinical chorioretinitis, cerebral edema with microhemorrhages, and bilateral central auditory pathway dysfunction underscores the critical importance of performing a comprehensive, multi-system evaluation in every infant diagnosed with CT, regardless of the apparent severity of initial symptoms. This case also highlights the real-world challenges of managing CT in regions where first-line medications are not readily accessible and supports the continued role of the pyrimethamine-sulfadiazine-folinic acid regimen as the recommended first-line therapy.

### 3.1 Epidemiology and risk factors in China

The incidence and disease burden of CT in China remain poorly defined due to the absence of mandatory prenatal or neonatal screening programs. A recent national cross-sectional study of hospitalized children with congenital TORCH infections in 27 Chinese children's hospitals from 2015 to 2020 found that CT accounted for only 5 of 173 cases (2.89%) but was associated with the longest median length of hospital stay (20 days), reflecting the complexity of managing this infection (5). The seroprevalence of *T. gondii* IgG among Chinese women of childbearing age is approximately 2.3%–5.0%, with higher rates observed in southwestern provinces such as Sichuan and Yunnan (16, 17). This regional variation is attributed to dietary habits. In our case, the mother resided in Hubei Province and reported contact with a cat but no consumption of undercooked meat; however, seroconversion during pregnancy was confirmed.

### 3.2 Vertical transmission and gestational timing

The risk of vertical transmission and the severity of fetal injury are inversely related to the gestational age at maternal seroconversion. Transmission rates are lowest in the first trimester (10%–15%) but are associated with the most severe fetal sequelae, including stillbirth and major neurological damage. In contrast, third-trimester infections have the highest transmission rates (>60%) but typically result in subclinical or mild disease at birth (8, 18). The combination of negative first-trimester maternal serology, postpartum maternal IgM/IgG positivity, neonatal IgM positivity, and repeated detection of T. gondii DNA in neonatal blood strongly supported congenital toxoplasmosis. This timing likely explains the relatively mild initial neonatal presentation with isolated jaundice, while detailed investigation revealed established CNS and ocular pathology. A recent multicenter Italian study by Bonetti et al. (19) also reported a substantial transmission risk following late-pregnancy maternal infection, a finding that closely aligns with our patient's clinical course.

### 3.3 Clinical spectrum and the importance of systematic screening

The classic triad of CT—chorioretinitis, intracranial calcifications, and hydrocephalus—is present in only a minority of symptomatic neonates (9). Jaundice, hepatosplenomegaly, rash, anemia, and thrombocytopenia are common but non-specific findings. Isolated jaundice, as seen in this case, has been reported in a handful of cases and can easily be misattributed to more common neonatal conditions such as ABO incompatibility or physiological jaundice (10). In the present case, common causes of neonatal hyperbilirubinemia, including immune hemolysis, G6PD deficiency, severe infection, hypothyroidism, and metabolic disease, were not supported clinically or laboratorily. Therefore, in any neonate with unexplained jaundice, especially when associated with maternal risk factors or early onset, a high index of suspicion for congenital infections, including toxoplasmosis, is warranted.

Once the diagnosis of CT is confirmed, a systematic evaluation for potential sequelae is mandatory, as subclinical involvement of the eyes, brain, and ears is common. In our patient, despite a normal physical examination and cranial CT, both fundoscopy and brain MRI revealed significant pathology. The RetCam examination disclosed active bilateral chorioretinitis, which, if left untreated, could progress to vision-threatening macular scarring or retinal detachment (20). The MRI demonstrated cerebral edema and microhemorrhages that were not visible on CT, highlighting the additional value of MRI for detecting early or subtle CNS involvement in CT (21).

### 3.4 Hearing impairment in congenital toxoplasmosis

Bilateral central auditory pathway dysfunction is an increasingly recognized but often underdiagnosed sequela of CT. A comprehensive literature review by Ferreira et al. (22) found a significant association between CT and both peripheral and central hearing impairment, as evidenced by abnormalities in BAEP and frequency-following response testing. The pathophysiology is multifactorial and may involve direct parasitic invasion of the cochlea and cochlear nerve, immune-mediated inflammation, and damage to central auditory pathways in the brainstem and cortex (23). Our patient's BAEP findings of prolonged I–V and III–V interpeak latencies are indicative of central auditory dysfunction, which is consistent with the observed cerebral edema on MRI. These findings underscore the importance of universal audiological screening in all infants with CT, even in the absence of overt neurological symptoms, to facilitate early intervention and optimize language development.

### 3.5 Diagnostic advances: The role of metagenomic next-generation sequencing

The diagnosis of CT traditionally relies on serological detection of *T. gondii*-specific IgM and IgA in neonatal serum, as IgG may be passively transferred from the mother (8). However, IgM assays can yield false-negative results, and confirmation by detection of parasite DNA in body fluids is recommended. Polymerase chain reaction (PCR) of amniotic fluid is the gold standard for prenatal diagnosis, while PCR of CSF, blood, or urine can aid in postnatal confirmation. In our case, the diagnosis was confirmed by blood mNGS, a highly sensitive, culture-independent molecular technique that allows for broad pathogen detection. The detection of T. gondii sequences on two separate occasions provided unequivocal evidence of active parasitemia. Interestingly, CSF mNGS was negative despite clinical and laboratory evidence of CNS inflammation. This discrepancy has been observed in other CNS infections and may be due to a low parasite burden in the CSF, the timing of lumbar puncture relative to the infection course, or sequestration of parasites within brain tissue (24). A recent study from El Salvador demonstrated that digital PCR may offer enhanced sensitivity for detecting perinatal T. gondii infection compared to conventional PCR (25), suggesting that more sensitive molecular tools may be required to definitively rule out CNS involvement in some cases. In resource-limited settings where targeted PCR assays are not routinely available, mNGS may provide supportive molecular evidence for congenital toxoplasmosis.

### 3.6 Therapeutic challenges and the evidence for alternative regimens

The management of this case was complicated by the initial unavailability of pyrimethamine, a situation that is not uncommon in many regions of China and other low- and middle-income countries. The actual dosing regimen used in this patient was determined after multidisciplinary consultation and was influenced by local drug availability and formulation accessibility. This forced a stepwise approach using alternative agents, providing a real-world illustration of their comparative efficacy.

Azithromycin: Azithromycin has shown activity against both tachyzoites and bradyzoites of T. gondii *in vitro* and in animal models (26). Several case reports, including one from China involving a severe case of CT with pancytopenia and splenomegaly, have described favorable outcomes with azithromycin monotherapy or combination therapy (14, 27). However, in our patient, 6 days of azithromycin monotherapy failed to improve CSF parameters, suggesting that it may have limited effectiveness as a sole agent in neonates with suspected CNS involvement.

Trimethoprim-Sulfamethoxazole (TMP-SMX): TMP-SMX is a fixed-dose combination antibiotic that inhibits folate synthesis in *T. gondii*. A network meta-analysis of treatments for ocular toxoplasmosis suggested that TMP-SMX may be an effective alternative to the standard regimen (15). However, its use in neonates under 2 months of age is generally contraindicated due to the risk of kernicterus from bilirubin displacement and bone marrow suppression. In our patient, after jaundice had resolved and with careful monitoring, a 13-day course of TMP-SMX combined with azithromycin was administered but did not lead to normalization of CSF pleocytosis. The limited response observed in this patient may have been related to multiple factors, including disease severity, treatment duration, or drug penetration into the CNS.

Pyrimethamine-Sulfadiazine-Folinic Acid: The rapid and sustained clinical, CSF, and radiological improvement observed after transitioning to the standard triple regimen further supports its continued role as the standard treatment for symptomatic CT (8, 11, 12). Pyrimethamine and sulfadiazine act synergistically to inhibit folate metabolism in the parasite, while folinic acid supplementation mitigates bone marrow toxicity in the host. The recommended treatment duration of 12 months is based on evidence that prolonged therapy reduces the risk of late-onset retinal disease and neurological sequelae (28). A recent study by Prasil et al. (29) comparing adverse reactions between spiramycin and pyrimethamine-sulfadiazine in pregnant women found that the latter was more effective and had a similar safety profile, further supporting its use.

### 3.7 Long-term prognosis and multidisciplinary follow-up

The long-term prognosis for infants with treated CT is generally favorable, but lifelong surveillance is necessary. Even with appropriate therapy, late-onset retinal lesions can occur in up to 30% of children, often presenting in adolescence or adulthood (20). Neurological sequelae, including cognitive impairment, seizures, and bilateral central auditory pathway dysfunction, may also develop or progress over time. Therefore, a structured, multidisciplinary follow-up program is essential. Our patient will be monitored regularly by pediatric infectious disease specialists, ophthalmologists (with dilated fundus examinations every 3–6 months), audiologists (with serial BAEP or behavioral audiometry), and developmental pediatricians. The decay of specific IgG antibody titers can be monitored to confirm successful clearance of the infection; a study by Salomè et al. (30) demonstrated that lower initial antibody titers in the mother-infant dyad predict earlier seronegativization, which can help guide the duration of follow-up.

### 3.8 Limitations

This case report has inherent limitations. The follow-up period is relatively short, and the patient's long-term visual, auditory, and neurodevelopmental outcomes remain to be determined. The absence of positive CSF molecular detection limits definitive microbiological confirmation of CNS infection, although the CSF inflammatory profile, neuroimaging abnormalities, and concurrent systemic evidence strongly supported CNS inflammatory involvement associated with congenital toxoplasmosis. Finally, the sequential use of different anti-parasitic regimens, necessitated by drug availability issues, introduces a confounding factor that limits our ability to draw firm conclusions about the efficacy of each individual agent. In addition, the sequential treatment modifications were driven primarily by local drug accessibility rather than a predefined comparative therapeutic strategy, limiting conclusions regarding comparative efficacy.

## 4 Conclusions

Congenital toxoplasmosis can present with subtle and non-specific findings, such as isolated neonatal jaundice. A high index of suspicion is required for timely diagnosis, particularly in regions with intermediate seroprevalence and no universal screening programs. This case highlights the critical importance of a systematic, multi-organ evaluation—including brain MRI, fundoscopy, and BAEP—in all infants diagnosed with CT, as subclinical ocular, cerebral, and auditory involvement is common. While alternative therapies like azithromycin and TMP-SMX may be considered when first-line agents are unavailable, they may provide limited short-term control in patients with suspected CNS involvement. The standard regimen of pyrimethamine, sulfadiazine, and folinic acid remains the recommended standard therapy and should be pursued whenever possible. Long-term, multidisciplinary follow-up is mandatory to detect and manage late-onset sequelae and optimize outcomes for affected children.

## Data Availability

The original contributions presented in the study are included in the article/Supplementary Material, further inquiries can be directed to the corresponding author/s.
